# Association of HBsAg Positivity and HBV Reactivation in *EGFR*-Mutant Lung Adenocarcinoma Treated with EGFR-TKIs: A Retrospective Cohort Study

**DOI:** 10.3390/cancers18203373

**Published:** 2026-10-18

**Authors:** Jiayi Sun, Jingwen Lin, Honglin Li, Weimin Li, Panwen Tian, Yalun Li

**Affiliations:** 1 Department of Pulmonary and Critical Care Medicine, West China Hospital, Sichuan University, Chengdu 610041, China; 2 State Key Laboratory of Respiratory Health and Multimorbidity, Institute of Respiratory Health and Multimorbidity, Institute of Respiratory Health, Frontiers Science Center for Disease-Related Molecular Network, Precision Medicine Center/Precision Medicine Key Laboratory of Sichuan Province, Sichuan University, Chengdu 610041, China; 3 Lung Cancer Center/Lung Cancer Institute, West China Hospital, Sichuan University, Chengdu 610041, China

**Keywords:** lung adenocarcinoma, EGFR-TKI, HBsAg positivity, hepatitis B virus, HBV reactivation

## Abstract

Background: EGFR-TKIs serve as standard first-line targeted therapy for *EGFR*-mutant lung adenocarcinoma (LUAD). Real-world data on the prognostic role of baseline HBsAg positivity and the risk of HBV reactivation during EGFR-TKI treatment remain limited, particularly in HBV-endemic Chinese populations. Methods: This retrospective single-center cohort study enrolled 580 consecutive patients with stage III–IV *EGFR*-mutant LUAD who received EGFR-TKIs between 2013 and 2026. Patients were grouped by baseline HBsAg status; Kaplan–Meier curves and multivariable Cox models adjusted for key clinical covariates were used to evaluate progression-free survival (PFS) and overall survival (OS). HBV reactivation was defined by EASL criteria, with virological evaluation available for 51 of 82 HBsAg-positive patients. Results: Among all enrolled patients, 14.1% were HBsAg-positive. The HBV reactivation rate was 9.8% among evaluable HBsAg-positive patients, and reactivation was not independently linked to survival. After adjustment, baseline HBsAg positivity showed a borderline trend toward poorer OS (HR = 1.42, 95% CI, 0.99–2.04, *p* = 0.054) without significant PFS association (HR = 1.14, 95% CI, 0.80–1.62, *p* = 0.481). Liver metastasis independently predicted worse OS in HBsAg-positive patients (HR = 3.84, 95% CI, 1.65–8.94, *p* = 0.002). Conclusions: HBV reactivation was uncommon but warrants close monitoring during EGFR-TKI therapy. Baseline HBsAg positivity demonstrated only a non-significant trend toward inferior OS without affecting PFS. Routine HBsAg screening at diagnosis may facilitate risk stratification, and HBsAg-positive patients with liver metastasis require intensified surveillance.

## Simple Summary

EGFR-targeted oral drugs are standard first-line treatment for advanced lung adenocarcinoma with *EGFR* gene mutations. In regions where hepatitis B virus (HBV) is common, like China, it remains unclear whether HBsAg positivity affects patient survival and how often HBV reactivation happens during targeted therapy. We studied 580 patients treated with this targeted treatment. We found that HBV reactivation was uncommon, and baseline HBsAg positivity only showed a borderline trend toward poorer long-term survival without affecting tumor progression. Among HBsAg-positive patients, liver metastasis predicts worse survival. These results highlight the need to identify high-risk patients at diagnosis to guide personalized care during EGFR-targeted therapy.

## 1. Introduction

Lung cancer represents the most common malignancy globally, and chronic hepatitis B virus (HBV) infection is highly endemic in China [1]. A 2022 modeling study reported that approximately 79.7 million individuals in China are living with chronic HBV infection, constituting a large population of HBV-infected individuals nationwide [2]. Although precise epidemiological estimates of patients with lung adenocarcinoma (LUAD) and concomitant chronic HBV infection remain unavailable, this population is likely substantial. HBV reactivation constitutes a critical clinical hazard during anti-tumor therapy in this co-infected cohort [3,4]. Previous studies have confirmed that among lung cancer patients with chronic HBV infection, chemotherapy and immune checkpoint inhibitors (ICIs) can trigger HBV reactivation, with reported incidences of 21% and 14.3%, respectively, in patients without prophylactic antiviral therapy [5,6,7]. Data on TKI-associated HBV reactivation remain scarce. With the exception of the TKI cohort reported by Yao et al. [8], most available publications consist of sporadic case reports of HBV reactivation or even life-threatening hepatic failure [9,10]. One recent real-world study further confirmed that baseline HBsAg positivity is an independent risk factor for HBV reactivation following targeted therapy [11]. Nevertheless, long-term prognostic data for this specific population remain insufficient, and the impact of baseline HBsAg status on survival outcomes in patients receiving EGFR-TKIs remains poorly defined. Furthermore, most randomized controlled trials (RCTs) of EGFR-TKIs excluded patients with chronic conditions such as chronic HBV infection, leading to a paucity of real-world evidence regarding EGFR-TKI efficacy and safety within this specific subgroup.

Accordingly, this study aimed to characterize clinical profiles and the incidence of HBV reactivation among HBsAg-positive patients with *EGFR*-mutant LUAD receiving EGFR-TKIs and to generate real-world evidence to guide clinical surveillance and prophylactic strategies for HBV reactivation in this population.

## 2. Materials and Methods

### 2.1. Study Design and Patient Selection

This retrospective cohort study included patients treated at West China Hospital, Sichuan University, with eligible cases identified from medical records covering the period January 2013 to January 2026. The inclusion criteria were histologically confirmed LUAD; stage III-IV disease defined by the 8th edition of the AJCC/UICC TNM staging system [12]; actionable *EGFR* mutations confirmed via next-generation sequencing (NGS) performed at the Center for Precision Medicine, West China Hospital, Sichuan University, using the NovaSeq 6000 platform (Illumina, San Diego, CA, USA), with clinical NGS panels selected per routine practice; receipt of EGFR-TKI treatment; and availability of complete baseline and follow-up clinical data. Patients were excluded if they had stage I-II disease, had enrolled in interventional clinical trials, harbored concurrent secondary malignancies, or had incomplete clinical or follow-up data.

**Figure 1:**
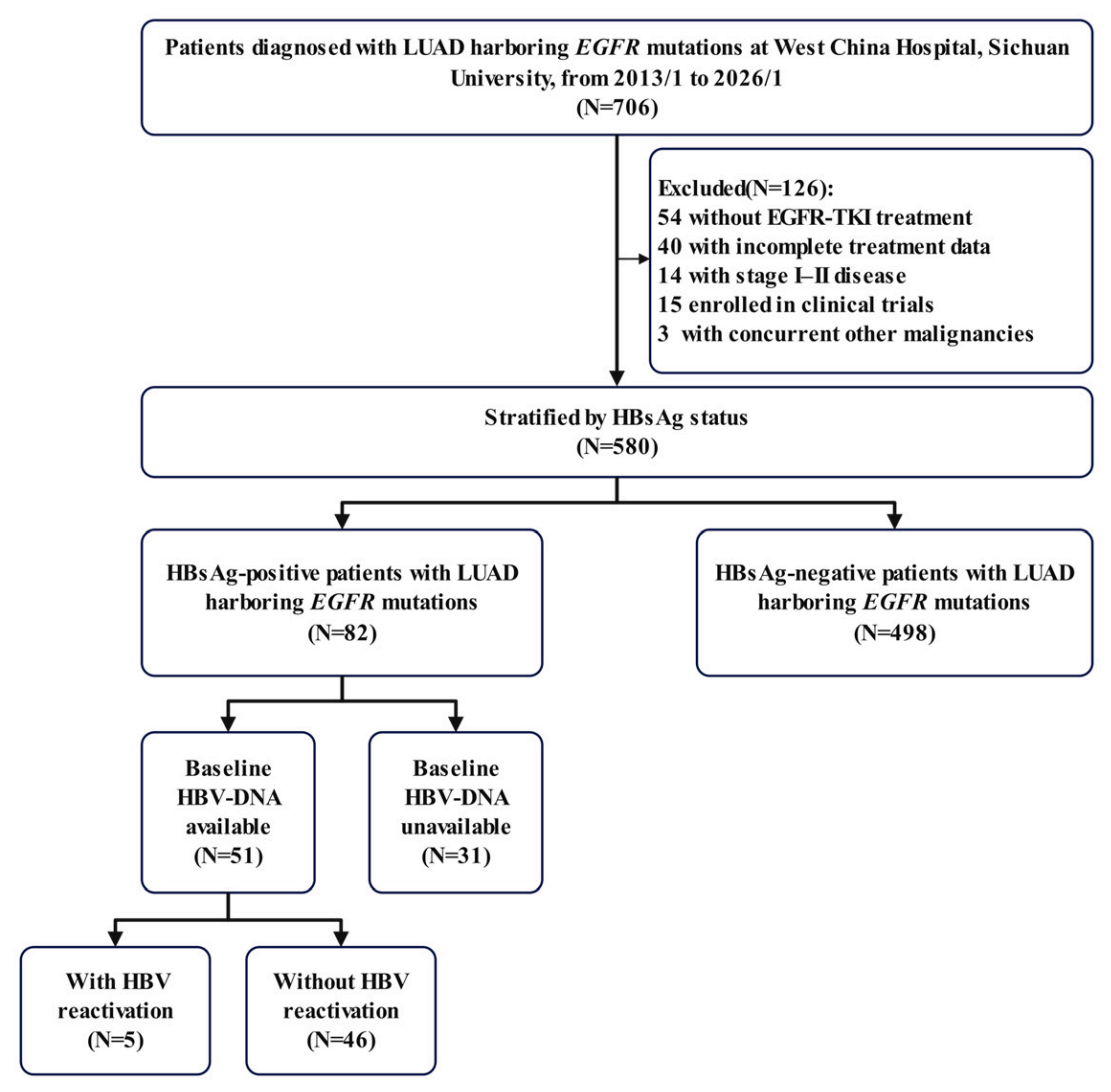
Patient selection and exclusion criteria.

A total of 706 patients were initially screened, and 126 ineligible patients were excluded, leaving 580 eligible patients stratified by baseline HBsAg serostatus (82 HBsAg-positive and 498 HBsAg-negative). Among the 82 HBsAg-positive patients, HBV reactivation evaluation was restricted to 51 patients with available baseline HBV-DNA data, of whom 5 experienced HBV reactivation during EGFR-TKI therapy. The remaining 31 HBsAg-positive patients without baseline HBV-DNA data were non-evaluable for HBV reactivation (Figure 1).

### 2.2. Clinical Data Collection

Baseline demographic and clinical data, including age, sex, smoking status, TNM stage, ECOG performance status, metastatic sites, treatment history, and tumor characteristics, were retrieved from electronic medical records. Laboratory parameters included liver function indices, HBsAg, HBeAg, anti-HBc, and baseline HBV-DNA levels. *EGFR* mutation subtypes, EGFR-TKI regimens, total treatment duration, and prophylactic antiviral administration were collected in detail.

Baseline HBsAg seropositivity was used to identify patients with chronic HBV carriage. HBV reactivation was defined according to European Association for the Study of the Liver (EASL) criteria: a ≥10-fold increase in HBV-DNA compared with baseline or newly detected HBV-DNA ≥100 IU/mL in patients with undetectable baseline viral load, with or without accompanying liver dysfunction [13].

### 2.3. Statistical Analysis

All statistical analyses were performed using R software (version 4.4.2). Categorical variables are presented as counts and percentages, while continuous variables are presented as median (interquartile range, IQR). Group differences in categorical variables were assessed using the χ^2^ test or Fisher’s exact test, as appropriate. Continuous variables were compared using the Mann–Whitney U test.

Missing data were handled via complete-case analysis. Twenty patients with unknown ECOG-PS status all belonged to the HBsAg-negative group, suggesting potential nonrandom missingness for this variable.

Overall survival (OS) and progression-free survival (PFS) were predefined as study endpoints (Section 2.4). Kaplan–Meier survival curves were generated, and between-group survival differences were compared using the log-rank test. Multivariable Cox proportional-hazards regression models were applied to identify independent prognostic factors for PFS and OS. A stratified Cox model was fitted for PFS (stratified by *EGFR* mutation subtype and EGFR-TKI generation), whereas a conventional non-stratified Cox model was used for OS, with *EGFR* mutation subtype and EGFR-TKI generation included as covariates (Table S1). All clinically meaningful covariates were pre-specified and incorporated into the models. The proportional-hazards assumption was validated using Schoenfeld residual tests (Table S2).

For analyses of both the overall cohort and the HBsAg-positive subgroup, multivariable Cox models were built using pre-specified covariates selected based on clinical plausibility and published evidence from cohorts of *EGFR*-mutant lung cancer and HBV-infected solid tumors. Covariate selection was not driven solely by univariate *p*-values. This pre-specification strategy was applied to minimize overfitting and selection bias, particularly for the relatively small HBsAg-positive subgroup (*n* = 82).

To assess the robustness of the primary findings regarding HBsAg status, a 1:1 nearest-neighbor propensity score matching (PSM) analysis without replacement was performed. Propensity scores were estimated using a logistic regression model incorporating the following pre-specified baseline covariates: age, sex, smoking status, family history of lung cancer, TNM stage, ECOG performance status, *EGFR* mutation subtype, EGFR-TKI generation, prior surgery, radiotherapy, chemotherapy alone, chemoimmunotherapy, liver metastasis, and TKI treatment line. Matches were carried out with a caliper width of 0.2 times the standard deviation of the logit of the propensity score. Standardized mean differences (SMDs) were calculated before and after matching to evaluate between-group balance; an SMD < 0.1 was considered to indicate adequate covariate balance. Residual imbalance in age (SMD > 0.1) persisted after matching; therefore, the Cox model within the PSM-matched cohort was additionally adjusted for age. After matching, survival outcomes between HBsAg-positive and HBsAg-negative patients were compared using Kaplan–Meier curves and multivariable Cox regression, as described above.

The primary hypothesis focused on the prognostic impact of baseline HBsAg status on OS and PFS. No formal multiplicity adjustment was performed for the secondary prognostic covariates incorporated into the multivariable models.

### 2.4. Follow-Up and Primary Endpoints

All patients received routine follow-up after EGFR-TKI initiation. Follow-up information was obtained through electronic medical record review and telephone interviews. The primary endpoint was OS, defined as the interval from EGFR-TKI initiation to all-cause death or the last follow-up date. The secondary endpoint was PFS, defined as the time from treatment initiation to radiological tumor progression or all-cause death, whichever occurred first. The data cutoff date was 31 January 2026.

The median follow-up duration for the entire cohort was 50.3 months, estimated by the reverse Kaplan–Meier method. During the observational period, 379 OS events and 470 PFS events were documented.

## 3. Results

### 3.1. Baseline Clinical Characteristics of Participants

We retrospectively enrolled 580 patients with *EGFR*-mutant LUAD treated with EGFR-TKIs. Patients were divided into 82 HBsAg-positive and 498 HBsAg-negative subgroups based on baseline HBsAg status. Baseline demographic, clinical, and molecular characteristics of the overall cohort and the two subgroups are summarized in Table 1.

Compared with HBsAg-negative patients, HBsAg-positive patients were significantly younger (median age, 54.0 years vs. 63.5 years; *p* < 0.001) and included a higher proportion of male patients (53.7% vs. 40.6%; *p* = 0.035). The HBsAg-positive subgroup had higher proportions of stage III disease and greater rates of prior surgery, radiotherapy, and chemotherapy. The two groups were well balanced in terms of ECOG performance status, smoking history, family history of lung cancer, *EGFR* mutation subtype, EGFR-TKI generation, prior chemoimmunotherapy, and liver metastasis (all *p* > 0.05). Baseline clinical characteristics of patients harboring uncommon *EGFR* mutations are summarized in Tables S3 and S4.

### 3.2. Survival Outcomes Stratified by HBsAg Status

**Figure 2:**
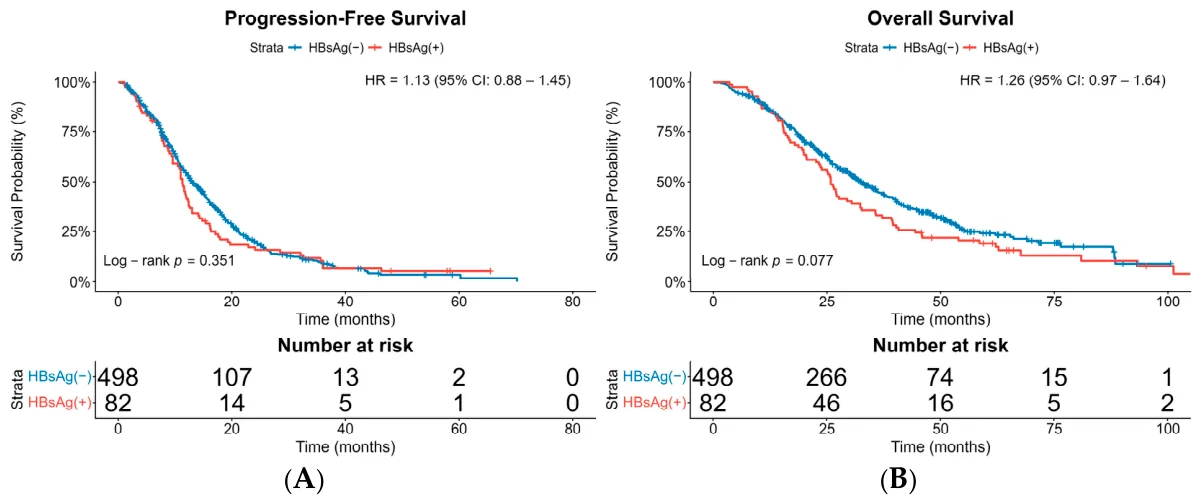
Kaplan–Meier survival curves stratified by HBsAg status: (**A**) progression-free survival; (**B**) overall survival. Log-rank tests were used to compare survival distributions between groups. Hazard ratios (HRs) and 95% confidence intervals (CIs) were derived from univariate Cox proportional-hazards regression. Number-at-risk tables are presented below each curve.

Kaplan–Meier survival curves were generated to compare PFS and OS between HBsAg-positive (*n* = 82) and HBsAg-negative (*n* = 498) patients. Median PFS was 11.3 months (95% CI, 9.6–12.4) among HBsAg-positive patients versus 13.0 months (95% CI, 12.0–14.8) in HBsAg-negative patients. Median OS was 25.9 months (95% CI, 23.3–32.4) versus 32.6 months (95% CI, 30.2–36.2), respectively. Survival distributions were compared using the log-rank test, while crude HRs and corresponding 95% CIs were estimated via univariate Cox proportional-hazards regression. PFS was comparable between the two groups (HR = 1.13, 95% CI, 0.88–1.45; Log-rank *p* = 0.351). HBsAg-positive patients exhibited a trend toward shorter OS, although this difference did not reach statistical significance (HR = 1.26, 95% CI, 0.97–1.64; Log-rank *p* = 0.077) (Figure 2).

### 3.3. Multivariable Cox Proportional-Hazards Regression Analysis

Multivariable Cox proportional-hazards regression models were used to identify independent prognostic factors for PFS and OS. After adjustment for clinically relevant confounders, HBsAg positivity showed a marginal trend toward shorter OS (HR = 1.423, 95% CI, 0.995–2.036; *p* = 0.054), whereas no significant independent association was detected between HBsAg status and PFS (HR = 1.14, 95% CI, 0.80–1.62; *p* = 0.481). In the PSM-matched cohort, the HR remained consistent in magnitude and direction (HR = 1.37, 95% CI, 0.85–2.22; *p* = 0.195), supporting the robustness of this trend, although statistical significance was not retained in the matched sample (Figures S1 and S2 and Table S5).

**Figure 3:**
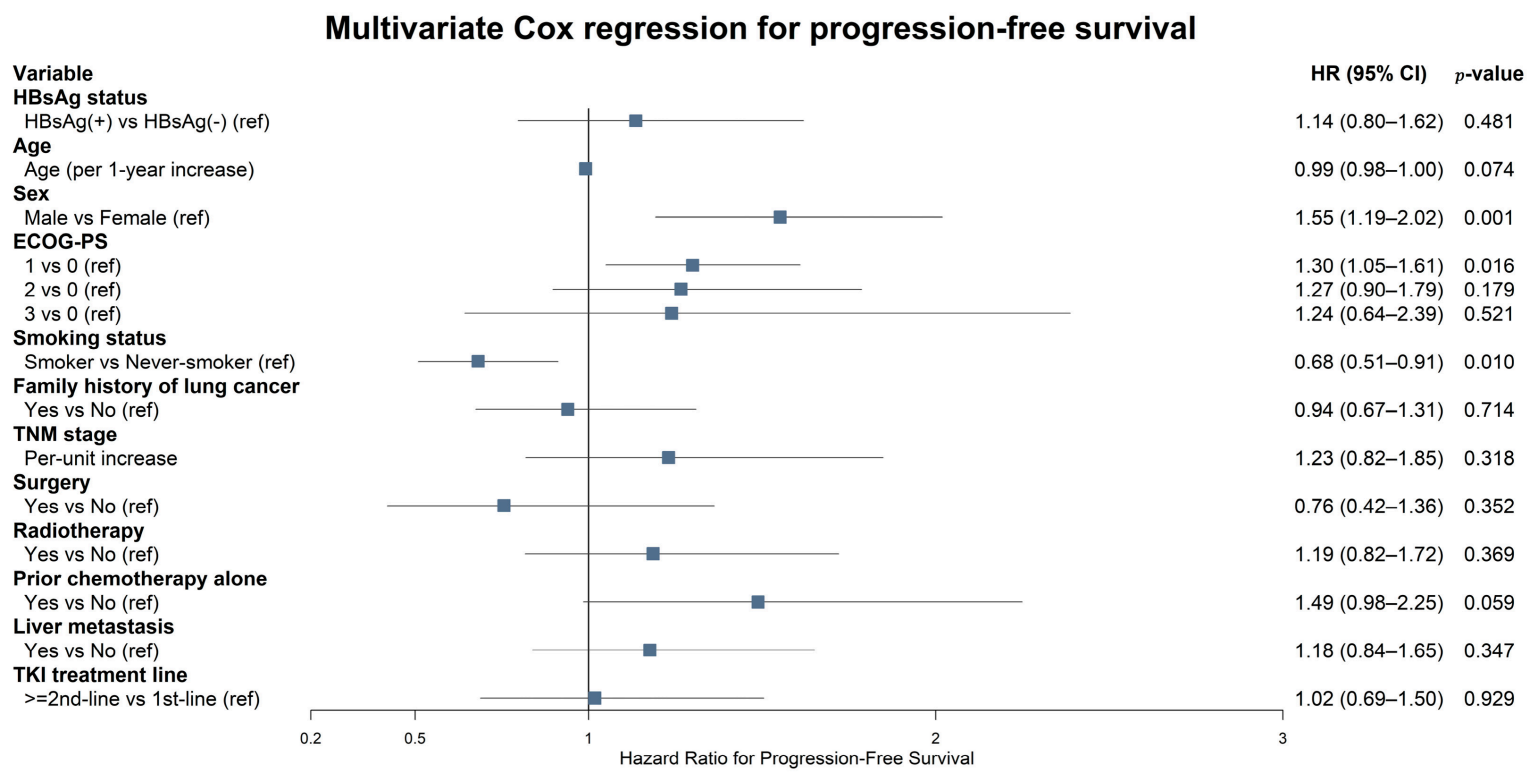
Multivariable Cox regression for progression-free survival. Stratified Cox proportional-hazards regression was applied, with stratification by *EGFR* mutation subtype and EGFR-TKI generation. TNM stage and TKI treatment line were included as covariates. The proportional-hazards assumption was verified using the global Schoenfeld residual test (χ^2^ = 16.230, df = 13; *p* = 0.237). Abbreviations: ref, reference group; HR, hazard ratio; CI, confidence interval.

**Figure 4:**
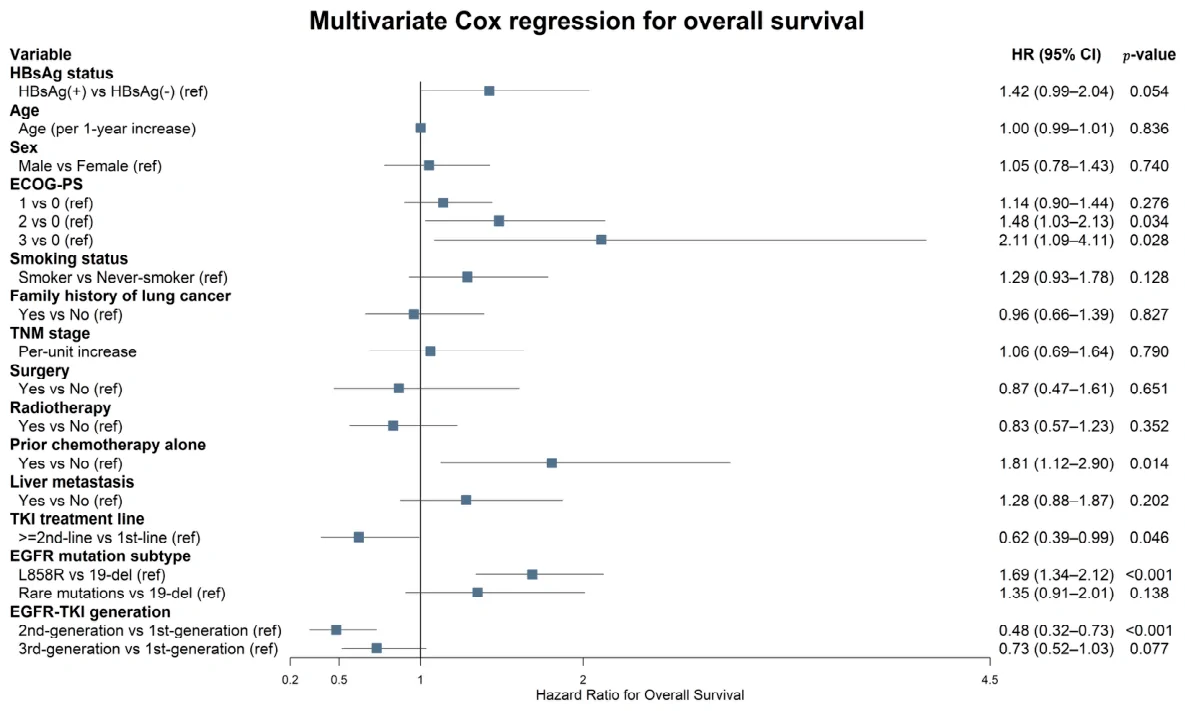
Multivariable Cox regression for overall survival. Multivariable Cox proportional-hazards regression was used to identify independent prognostic factors for OS. The TKI treatment line was included as a covariate. The proportional-hazards assumption was verified using the global Schoenfeld residual test (χ^2^ = 14.615, df = 18; *p* = 0.688). Abbreviations: ref, reference group; HR, hazard ratio; CI, confidence interval.

For PFS, male sex (HR = 1.55, 95% CI, 1.19–2.02; *p* = 0.001) and elevated ECOG performance status (ECOG 1 vs. 0; HR = 1.30, 95% CI, 1.05–1.61; *p* = 0.016) were independent adverse prognostic factors for progression. Notably, smoking history (HR = 0.68, 95% CI, 0.51–0.91; *p* = 0.010) emerged as an independent protective factor (Figure 3). For OS, poorer ECOG performance status (ECOG 3 vs. 0; HR = 2.11, 95% CI, 1.09–4.11; *p* = 0.028), L858R mutation (vs exon 19 deletion; HR = 1.69, 95% CI, 1.34–2.12; *p* < 0.001), and second-generation EGFR-TKI therapy (HR = 0.48, 95% CI, 0.32–0.73; *p* < 0.001) were significantly associated with OS (Figure 4). Exploratory interaction P values for HBsAg status across prespecified clinical subgroups are shown in Table S6.

We calculated the events-per-variable (EPV) ratio to assess model stability. In the overall cohort, the EPV was 18.95 for the OS multivariable model and 36.15 for the PFS stratified Cox model. Within the HBsAg-positive subgroup multivariable Cox models, 75 PFS events and 73 OS events were observed, yielding EPV ratios of 7.5 for the PFS model and 6.64 for the OS model. Post hoc power analysis for the primary HBsAg status comparison showed a minimal detectable HR of 1.511 for OS and 1.449 for PFS (80% power, two-sided α = 0.05).

### 3.4. HBV Reactivation Events in HBsAg-Positive Patients

**Figure 5:**
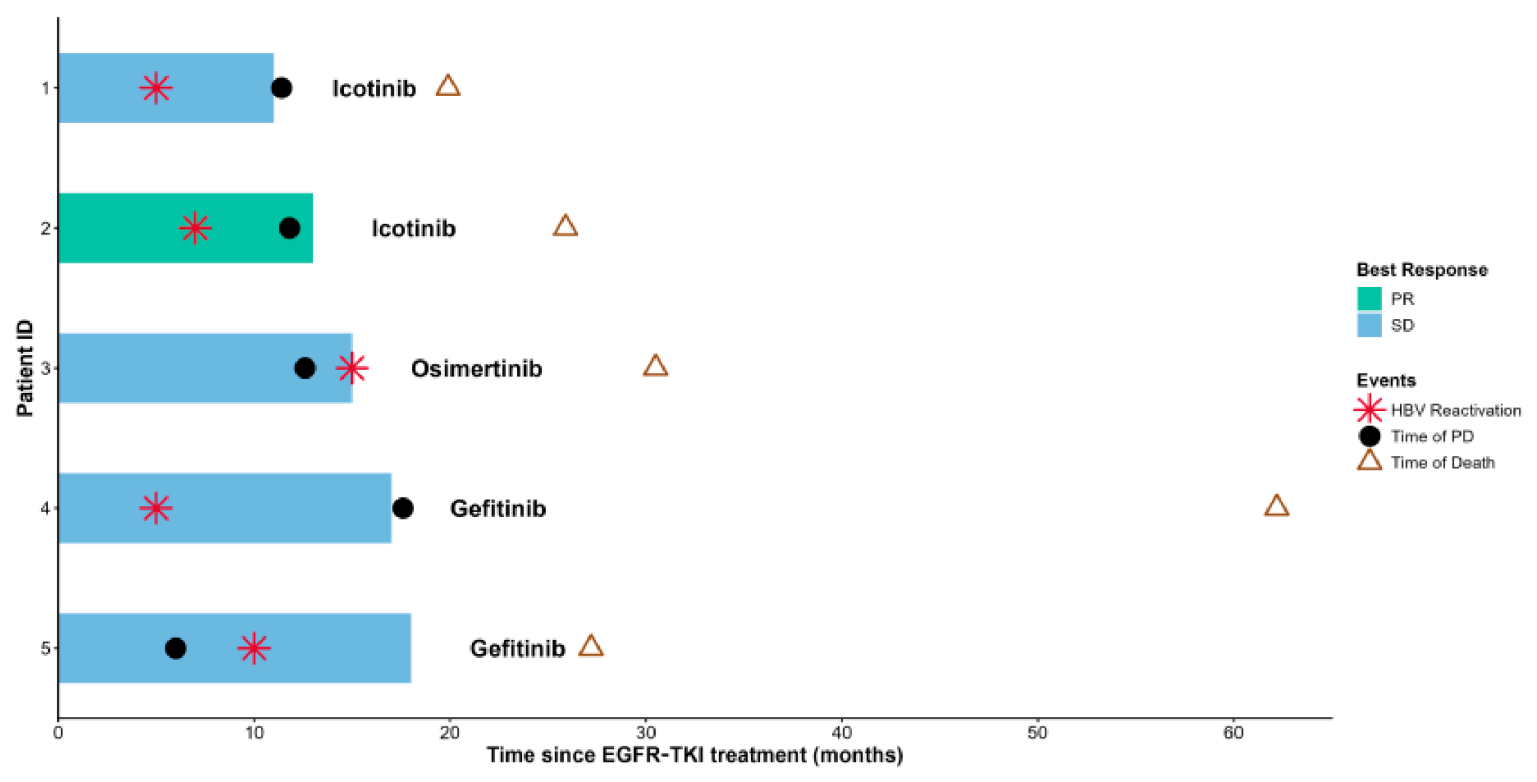
Swimmer plot illustrating treatment courses of five patients who experienced HBV reactivation during EGFR-TKI therapy. Horizontal bars represent the duration of EGFR-TKI treatment. Red asterisk: time point of HBV reactivation; black circle: progressive disease; open triangle: death. PR, partial response; SD, stable disease.

Among the 82 HBsAg-positive patients treated with EGFR-TKI therapy, baseline HBV-DNA data were available for 51 individuals, whereas 31 patients lacked baseline HBV-DNA measurements. A baseline comparison between patients with available and missing HBV-DNA data is presented in Table S7. Given the non-random pattern of HBV-DNA missingness suggested by several imbalanced baseline variables, we performed best-case–worst-case sensitivity analysis. Under the best-case scenario assuming no reactivation among patients with missing data, the overall HBV reactivation rate was 6.1% (5/82). Under the worst-case scenario assuming all patients with missing baseline HBV-DNA developed HBV reactivation, the rate rose to 43.9% (36/82). This wide range reflects considerable uncertainty in the true incidence of HBV reactivation in the full HBsAg-positive cohort. HBV reactivation occurred in 5 of these 51 patients, corresponding to an incidence of 9.8%. Detailed individual clinical–virological characteristics, antiviral management, and survival outcomes of the five reactivation cases are summarized in Table S8, and their longitudinal treatment trajectories are visualized in Figure 5.

Briefly, HBV reactivation occurred 5 to 15 months after EGFR-TKI initiation, with 2 cases under icotinib, 2 under gefitinib, and 1 under osimertinib. Two patients developed reactivation despite prophylactic entecavir, and all 5 cases showed a marked rise in HBV-DNA after reactivation (peak: 183-23500 IU/mL). Of these, 4 patients had low/undetectable baseline HBV-DNA, and 1 had a high baseline viral load (3780 IU/mL). Notably, four of these five patients had high baseline HBsAg levels (>3000 or 5042 IU/mL), while only one patient had a relatively low baseline HBsAg of 658 IU/mL. Four patients experienced varying degrees of ALT elevation during reactivation, while only 1 patient had normal liver function throughout. The best tumor response was partial response (PR) in 1 patient and stable disease (SD) in the remaining 4. Post-reactivation management included adding or continuing nucleos(t)ide analogues, with 1 patient treated with no additional antiviral intervention.

### 3.5. Prognostic Factors Among HBsAg-Positive Patients

Within the HBsAg-positive subgroup (*n* = 82), univariate and multivariable Cox regression analyses were performed to identify prognostic factors for PFS and OS (Tables S9 and S10).

In the multivariable PFS model, male sex showed a marginally elevated risk of disease progression (HR = 1.64, 95% CI, 0.98–2.77, *p* = 0.061). No statistically significant independent associations were observed for EGFR-TKI generation, TNM stage, HBV reactivation, or TKI treatment line in relation to PFS.

For OS, liver metastasis was an independent adverse prognostic factor associated with increased risk of death (HR = 3.84, 95% CI, 1.65–8.94, *p* = 0.002). Use of second-generation EGFR-TKIs was independently associated with reduced death risk compared with first-generation EGFR-TKIs (HR = 0.28, 95% CI, 0.09–0.83, *p* = 0.021). TNM stage, HBV reactivation, prior antiviral regimens, and TKI treatment line were not significantly associated with OS after full adjustment. All multivariable analyses in this HBsAg-positive subgroup were exploratory, and no adjustment for multiple testing was performed.

## 4. Discussion

In this retrospective cohort of patients with *EGFR*-mutant LUAD treated with EGFR-TKI therapy, we primarily evaluated the prognostic impact of baseline HBsAg positivity, with secondary exploratory analyses of on-treatment HBV reactivation restricted to the HBsAg-positive subgroup. Of note, the adverse prognostic effect of HBsAg positivity on OS was non-significant in univariate analysis (HR = 1.26, *p* = 0.077), and only approached marginal significance after multivariate adjustment for baseline covariates (HR = 1.423, *p* = 0.054). This finding indicates negative confounding: HBsAg-positive patients exhibited more favorable baseline characteristics, including younger age and higher rates of surgical resection. These baseline advantages attenuated the crude association between chronic HBV infection and survival observed in univariate comparisons. Prior studies have demonstrated that HBV infection correlates with survival in lung cancer patients treated with chemotherapy, whereas its prognostic significance remains inconsistent among patients treated with immune-checkpoint inhibitors [6,14,15]. Our findings add real-world evidence specific to the EGFR-TKI targeted-therapy setting, showing a trend toward worse outcomes associated with chronic HBV carriage for this patient population. Within the HBsAg-positive subgroup, liver metastasis was an independent adverse prognostic factor for OS (HR = 3.84, *p* = 0.002). This observation is consistent with previous real-world reports from unselected *EGFR*-mutant NSCLC cohorts, in which liver metastasis was established as a strong adverse prognostic marker under EGFR-TKI therapy [16,17,18]. Beyond intrinsic tumor aggressiveness, liver metastasis remodels the hepatic immune microenvironment, potentially disrupting intrahepatic immune surveillance against HBV and further worsening clinical outcomes among HBV carriers [19,20].

After adjustment for confounders, HBV reactivation during EGFR-TKI therapy showed no independent association with either PFS or OS among HBsAg-positive patients. Of the 82 HBsAg-positive patients in our cohort, only 51 had measurable baseline HBV-DNA for reactivation assessment, among whom 5 developed HBV reactivation, corresponding to an incidence of 9.8%. Reactivation occurred in 2 patients treated with antiviral prophylaxis and 3 patients without prophylaxis. This incidence was comparable to the 9.36% reported by Yao et al. [8]. A large Korean nationwide TKI cohort likewise identified HBsAg positivity as the only independent predictor of HBV reactivation in lung cancer patients receiving TKIs, with a markedly increased HR, supporting our risk stratification [11]. Although no deaths attributable to HBV-related liver failure were observed in our cohort, severe and even life-threatening hepatic injury secondary to TKI-triggered HBV reactivation has been well-documented [21]. Importantly, patients with resolved HBV infection (anti-HBc-positive, HBsAg-negative) are also at risk of viral reactivation under targeted therapy [10], reinforcing the need for regular virological surveillance and long-term risk management for all HBV-exposed individuals. Given the absence of uniform serial HBV-DNA monitoring among HBsAg-negative subjects, we cannot determine the actual risk of HBV reactivation in patients with resolved HBV infection (anti-HBc-positive, HBsAg-negative) treated with EGFR-TKIs. This represents an important study limitation and does not exclude the potential risk of HBV reactivation in this population.

Notably, in the multivariable stratified Cox model for PFS, smoking status was associated with a nominally lower risk of disease progression (HR = 0.68, 95% CI, 0.51–0.91, *p* = 0.010). This apparently protective association is biologically counterintuitive for lung cancer [22]. In a large systematic review and meta-analysis of EGFR-TKI randomized trials, smoking was reported as an adverse prognostic factor for PFS in *EGFR*-mutated NSCLC (pooled HR = 1.26, 95% CI, 1.05–1.51) [23]. This counterintuitive finding likely stems from residual confounding and inherent selection bias unique to this cohort of patients with *EGFR*-mutant lung adenocarcinoma. We emphasize that this result should only be regarded as an incidental, hypothesis-generating observation and must not be interpreted as evidence supporting a causal protective effect of smoking.

The exact mechanisms underlying HBV reactivation under EGFR-TKI treatment remain incompletely elucidated. Distinct HBV reactivation rates across chemotherapy, ICIs, and targeted agents are largely attributed to divergent drug-specific immunomodulatory properties. Sustained anti-HBV immune surveillance relies on functional T- and B-cell responses [24]. TKIs can interfere with intracellular kinase signaling, impair hepatocyte intrinsic antiviral defense, and compromise host immune surveillance. Among TKIs, imatinib is the best-characterized agent for promoting HBV reactivation: it inhibits c-Abl kinase, reduces degradation of HBV polymerase, and enhances cccDNA-driven viral transcription, thereby awakening latent HBV [25]. Preclinical data have shown that erlotinib suppresses T-cell proliferation, alters Th1/Th2 cytokine profiles, and induces T-cell anergy [26], which may disrupt anti-HBV immune homeostasis and facilitate cccDNA activation and viral rebound. However, one preclinical study has reported the opposite biological effect: erlotinib and gefitinib can directly suppress HBV replication by downregulating STAT3 phosphorylation in hepatocytes and attenuating cccDNA-dependent viral transcription [27]. Afatinib has also been reported to exert transient immunosuppressive effects through off-target inhibition of CAD, a rate-limiting enzyme of de novo pyrimidine biosynthesis, which suppresses CD8^+^ T-cell proliferation [28]. Whether similar immune perturbations apply to other generations of EGFR-TKIs warrants further investigation.

In our cohort, 41 out of 82 HBsAg-positive patients (50.0%) received antiviral prophylaxis, with entecavir (ETV, 32.9%) as the most frequently prescribed agent, followed by tenofovir disoproxil fumarate (TDF, 12.2%). Prior lamivudine exposure appeared correlated with higher risk of disease progression; however, this observation must be interpreted with great caution because of the small sample size and potential risk of bias. Consistent with current AASLD guidelines, lamivudine is not recommended for HBV reactivation prophylaxis due to high resistance risk [29]. Existing literature regarding the protective effect of antiviral prophylaxis in this setting remains conflicting. A meta-analysis by Su et al. reported no significant association between prophylactic antiviral therapy and reduced HBV reactivation risk [30], while Lee et al. demonstrated that antiviral prophylaxis effectively lowers the risk of HBV reactivation in HBsAg-positive lung cancer patients receiving TKIs [11]. Current AGA guidelines stratify the risk of TKI-associated HBV reactivation according to HBV serostatus; HBsAg-positive patients are categorized as high-risk individuals. This risk stratification is primarily derived from data on TKIs, including imatinib and sunitinib [31]. In our study, the number of observed HBV reactivation events was limited. Furthermore, two breakthrough HBV reactivation cases occurred among patients treated with prophylactic entecavir (ETV). The limited number of reactivation events, together with the occurrence of breakthrough reactivation, restricted the statistical power to detect a definite protective effect of prophylactic therapy. Therefore, larger, standardized cohorts are required to clarify the clinical value of antiviral prophylaxis in chronic HBV carriers treated with EGFR-TKI therapy.

Notably, substantial heterogeneity exists in the definition of HBV reactivation across studies and clinical guidelines, leading to varied reported incidence. We adopted the internationally recognized EASL criteria to define HBV reactivation: a ≥10-fold rise in HBV-DNA from baseline, or HBV-DNA ≥100 IU/mL in patients with undetectable baseline viral load, with or without liver dysfunction [13]. However, other guidelines set stricter viral thresholds or require concurrent transaminitis. The 2018 AASLD guideline defines virological reactivation as a ≥1 log10 IU/mL increase in HBV-DNA or an absolute level ≥1000 IU/mL, while clinical reactivation requires obvious ALT elevation [29]. The 2021 APASL guideline applies a cutoff of ≥2000 IU/mL for baseline HBV-DNA-negative patients, combined with liver dysfunction for comprehensive evaluation [32]. These definitional inconsistencies hamper cross-study comparisons and partially account for the broad range of HBV reactivation rates reported in the literature. Given the lack of a globally agreed standard, unified diagnostic criteria for HBV reactivation in patients treated with EGFR-TKIs are urgently required to standardize risk stratification and clinical management.

## 5. Limitations

This study has several key limitations. First, the single-center retrospective design carries inherent risks of selection bias and unmeasured confounding. Although multivariable Cox regression mitigated baseline imbalances between HBsAg subgroups, residual confounding cannot be fully excluded. Second, the small size of the HBsAg-positive subgroup and the limited number of HBV reactivation events may reduce statistical power for subgroup analyses exploring antiviral-related factors and predictors of HBV reactivation. In addition, multivariable Cox models in the HBsAg-positive subgroup yielded EPV values below the conventional threshold of 10 (6.64 for OS and 7.5 for PFS), which may result in unstable model coefficients and wide confidence intervals. Accordingly, prognostic estimates derived from this exploratory subgroup model should be interpreted with caution. Virological monitoring frequency during anti-cancer treatment was not standardized across patients, which may affect the ascertainment of HBV-related events. Moreover, we did not specifically capture liver-specific endpoints, such as hepatitis flare and liver-related mortality. Third, incomplete baseline HBV-DNA data precluded a robust assessment of the prognostic role of baseline viral load. Furthermore, we lacked granular data, including liver injury severity, detailed antiviral regimens, HBV genotypes, and immune dynamics, which prevented further mechanistic exploration of HBV-reactivation-associated outcomes.

## 6. Conclusions

In conclusion, among HBsAg-positive patients with *EGFR*-mutant LUAD treated with EGFR-TKI therapy, the HBV reactivation rate was 9.8% among patients with evaluable baseline HBV-DNA data. HBV reactivation was not independently associated with survival, although this analysis was limited by the small number of events. Baseline HBsAg positivity showed only a marginal trend toward poorer OS without a significant impact on PFS. For HBsAg-positive patients, intensified surveillance is recommended, particularly for those with liver metastasis. Routine HBsAg screening at initial diagnosis supports risk stratification and informs tailored clinical management during EGFR-TKI treatment.

## Figures and Tables

**Table 1:** Baseline clinical characteristics of patients with *EGFR*-mutant lung adenocarcinoma stratified by baseline HBsAg status.

Variable	Overall (*N* = 580)	HBsAg(+) (*n* = 82)	HBsAg(−) (*n* = 498)	*p*-Value
Age, median [IQR]	62.0 [52.8, 69.8]	54.0 [48.0, 61.0]	63.5 [54.1, 71.0]	<0.001
Sex, *n* (%)				0.035
Female	334 (57.6)	38 (46.3)	296 (59.4)	
Male	246 (42.4)	44 (53.7)	202 (40.6)	
ECOG performance status, *n* (%)				0.106
0	274 (47.2)	48 (58.5)	226 (45.4)	
1	213 (36.7)	27 (32.9)	186 (37.3)	
2	58 (10.0)	6 (7.3)	52 (10.4)	
3	15 (2.6)	1 (1.2)	14 (2.8)	
Unknown	20 (3.4)	0 (0.0)	20 (4.0)	
Smoking status, *n* (%)				0.373
Never-smoker	411 (70.9)	55 (67.1)	356 (71.5)	
Smoker	163 (28.1)	27 (32.9)	136 (27.3)	
Unknown	6 (1.0)	0 (0.0)	6 (1.2)	
Lung cancer family history, *n* (%)				0.254
No	513 (88.4)	76 (92.7)	437 (87.8)	
Yes	54 (9.3)	6 (7.3)	48 (9.6)	
Unknown	13 (2.2)	0 (0.0)	13 (2.6)	
*EGFR* mutation type ^a^, *n* (%)				0.267
Exon 19 deletion	274 (47.2)	43 (52.4)	231 (46.4)	
L858R	231 (39.8)	25 (30.5)	206 (41.4)	
Rare mutations	55 (9.5)	10 (12.2)	45 (9.0)	
Unknown subtype	20 (3.4)	4 (4.9)	16 (3.2)	
EGFR-TKI generation ^b^, *n* (%)				0.434
First-generation	408 (70.3)	56 (68.3)	352 (70.7)	
Second-generation	71 (12.2)	8 (9.8)	63 (12.7)	
Third-generation	101 (17.4)	18 (22.0)	83 (16.7)	
TNM stage, *n* (%)				0.010
III	52 (9.0)	14 (17.1)	38 (7.6)	
IV	528 (91.0)	68 (82.9)	460 (92.4)	
Surgery, *n* (%)				<0.001
Yes	22 (3.8)	19 (23.2)	3 (0.6)	
No	558 (96.2)	63 (76.8)	495 (99.4)	
Radiotherapy, *n* (%)				<0.001
Yes	69 (11.9)	47 (57.3)	22 (4.4)	
No	511 (88.1)	35 (42.7)	476 (95.6)	
Prior chemotherapy alone, *n* (%)				0.005
Yes	97 (16.7)	23 (28.0)	74 (14.9)	
No	483 (83.3)	59 (72.0)	424 (85.1)	
Prior chemoimmunotherapy, *n* (%)				0.067
Yes	26 (4.5)	0 (0.0)	26 (5.2)	
No	554 (95.5)	82 (100.0)	472 (94.8)	
TKI line, *n* (%)				0.314
First-line	468 (80.7)	70 (85.4)	398 (79.9)	
Second-line	112 (19.3)	12 (14.6)	100 (20.1)	
Liver metastasis, *n* (%)				0.632
Yes	52 (9.0)	9 (11.0)	43 (8.6)	
No	528 (91.0)	73 (89.0)	455 (91.4)	

Continuous variables are presented as median (IQR), and categorical variables are expressed as n (%). Between-group comparisons were performed using the Mann–Whitney U test for continuous variables and the χ^2^ test or Fisher’s exact test for categorical variables, as appropriate. Abbreviations: ECOG, Eastern Cooperative Oncology Group; EGFR, epidermal growth factor receptor; HBsAg, hepatitis B surface antigen; IQR, interquartile range; TKI, tyrosine kinase inhibitor. ^a^ *EGFR* mutation types were categorized into four groups: exon 19 deletion, L858R mutation, other rare *EGFR* mutations, and unclassified mutations. Other rare *EGFR* mutations included G719 family mutations (G719X/G719A, including concurrent G719 and S768I, *n* = 17), 19delins (*n* = 15), L861Q (*n* = 12), primary T790M (*n* = 3), isolated S768I *(n* = 3), E709A/E709Q (*n* = 2), 18delinsD (*n* = 1), F712S (*n* = 1), and L833V (*n* = 1). Unclassified mutations were defined as confirmed *EGFR* mutations without specific subtype information documented in the medical records (Tables S3 and S4). ^b^ First-generation EGFR-TKIs included gefitinib, erlotinib, and icotinib; second-generation EGFR-TKIs included afatinib and dacomitinib; third-generation EGFR-TKIs included osimertinib, avitinib, and furmonertinib.

## Data Availability

The data that support the findings of this study are available from the corresponding author upon reasonable request. Restrictions apply to the availability of these data due to patient privacy and ethical requirements.

## Supplementary Materials

The following supporting information can be downloaded at https://www.mdpi.com/article/10.3390/cancers18203373/s1, Figure S1: Kaplan–Meier overall survival curves stratified by baseline HBsAg status; Figure S2: Forest plot of hazard ratios for overall survival associated with HBsAg positivity across three analytical models; Table S1: Multivariate Cox regression results for overall survival and progression-free survival; Table S2: Proportional-hazards assumption test based on Schoenfeld residuals for Cox regression models; Table S3: Baseline clinical characteristics of patients with primary T790M, L861Q, and G719 family *EGFR* mutations; Table S4: Baseline clinical characteristics of patients with other uncommon *EGFR* mutations, including exon 19 insertions and isolated S768I; Table S5: Standardized mean differences (SMDs) of baseline covariates before and after propensity score matching; Table S6: Exploratory interaction *p*-values for HBsAg status with pre-specified clinical covariates in multivariate Cox regression models for overall survival and progression-free survival; Table S7: Baseline characteristics of HBsAg-positive *EGFR*-mutant NSCLC patients stratified by HBV-DNA test availability; Table S8: Clinical characteristics and management of HBV reactivation in *EGFR*-mutant lung adenocarcinoma patients treated with EGFR-TKI; Table S9: Univariate Cox regression analyses for progression-free survival and overall survival in HBsAg-positive patients; Table S10: Multivariable Cox regression analyses for progression-free survival and overall survival in HBsAg-positive patients.

## Abbreviations

The following abbreviations are used in this manuscript:

AASLD	American Association for the Study of Liver Diseases
AJCC	American Joint Committee on Cancer
ALT	Alanine transaminase
APASL	Asian Pacific Association for the Study of the Liver
cccDNA	Covalently closed circular DNA
CI	Confidence interval
EASL	European Association for the Study of the Liver
ECOG	Eastern Cooperative Oncology Group
EPVs	Events per variable
ETV	Entecavir
EGFR	Epidermal growth factor receptor
HBV	Hepatitis B virus
HBsAg	Hepatitis B surface antigen
anti-HBc	Hepatitis B core antibody
HR	Hazard ratio
ICI	Immune checkpoint inhibitor
IQR	Interquartile range
LUAD	Lung adenocarcinoma
NGS	Next-generation sequencing
OS	Overall survival
PFS	Progression-free survival
PR	Partial response
PSM	Propensity score matching
RCT	Randomized controlled trial
SD	Stable disease
SMD	Standardized mean difference
TDF	Tenofovir disoproxil fumarate
TKI	Tyrosine kinase inhibitor
TNM	Tumor, node, metastasis
UICC	Union for International Cancer Control
