# Supplementary material for: Association of HBsAg Positivity and HBV Reactivation in EGFR-Mutant Lung Adenocarcinoma Treated with EGFR-TKIs: A Retrospective Cohort Study
Source: Cancers (Basel). 2026 Oct 18;18(20):3373. doi: 10.3390/cancers18203373 (PMC13702524; doi:10.3390/cancers18203373)
Supplement: Supplementary file 1 [file cancers-18-03373-s001.zip › cancers-4608891-supplementary.pdf]

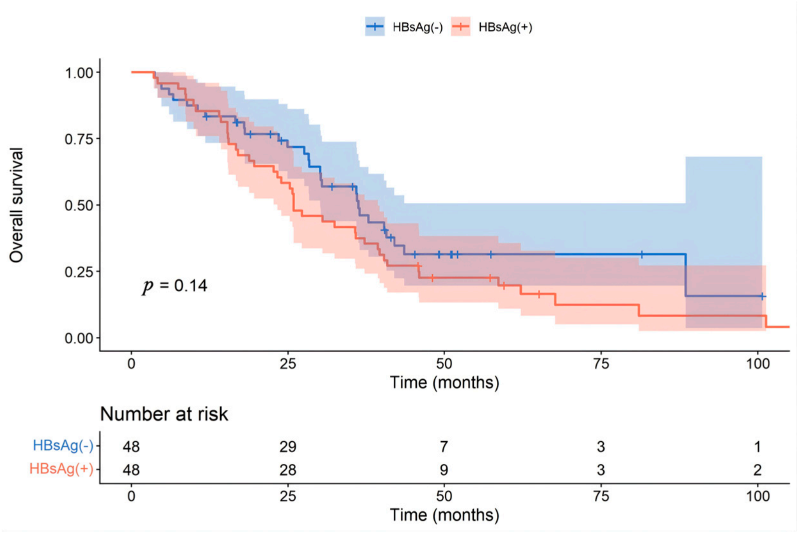

Supplementary Figure S1. Kaplan–Meier overall survival curves stratified by baseline HBsAg status (HBsAg-positive vs. HBsAg-negative).

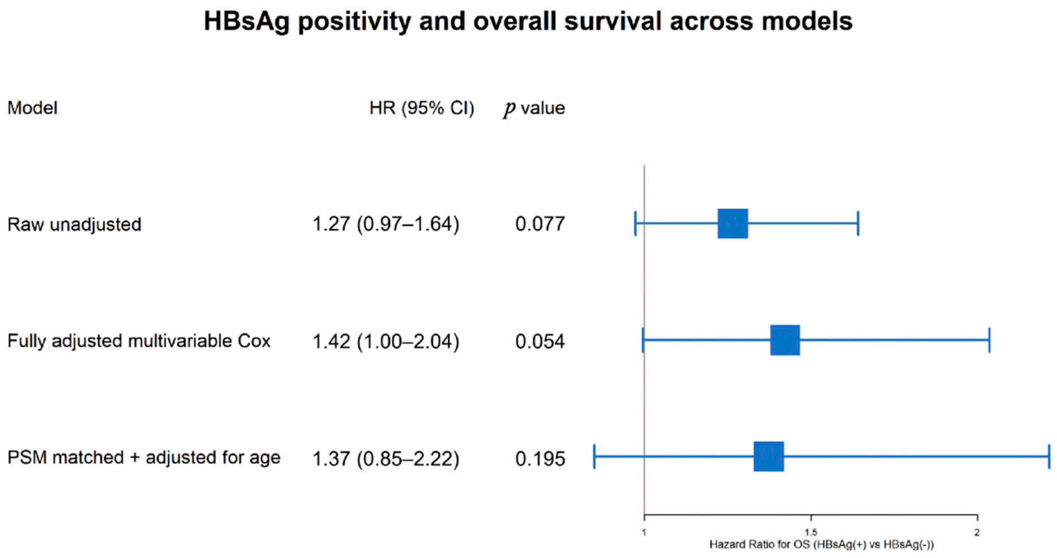

Supplementary Figure S2. Forest plot of hazard ratios for overall survival associated with HBsAg positivity across three analytical models.

Supplementary Table S1. Multivariate Cox regression results for overall survival and progression-free survival

Covariates	OS-Standard Cox		PFS-Stratified Cox[1]	
	**HR (95% CI)**	***p*-value**	**HR (95% CI)**	***p*-value**
HBsAg status (HBsAg+ vs HBsAg−)	1.42 (0.99–2.04)	0.054	1.14 (0.80–1.62)	0.481
Age (per 1-year increase)	1.00 (0.99–1.01)	0.836	0.99 (0.98–1.00)	0.074
Sex (Male vs Female)	1.05 (0.78–1.43)	0.740	1.55 (1.19–2.02)	0.001
ECOG-PS				
1 vs 0	1.14 (0.90–1.44)	0.276	1.30 (1.05–1.61)	0.016
2 vs 0	1.48 (1.03–2.13)	0.034	1.27 (0.90–1.79)	0.179
3 vs 0	2.11 (1.09–4.11)	0.028	1.24 (0.64–2.39)	0.521
Smoking status (Smoker vs Never-smoker)	1.29 (0.93–1.78)	0.128	0.68 (0.51–0.91)	0.010
Lung cancer family history (Yes vs No)	0.96 (0.66–1.39)	0.827	0.94 (0.68–1.31)	0.714
TNM stage (Per-unit increase)	1.06 (0.69–1.64)	0.790	1.23 (0.82–1.85)	0.318
Surgery (Yes vs No)	0.87 (0.47–1.61)	0.651	0.76 (0.42–1.36)	0.352
Radiotherapy (Yes vs No)	0.83 (0.57–1.23)	0.352	1.19 (0.82–1.72)	0.369
Prior chemotherapy alone (Yes vs No)	1.81 (1.13–2.90)	0.014	1.49 (0.98–2.25)	0.059
Liver metastasis (Yes vs No)	1.28 (0.88–1.87)	0.202	1.18 (0.84–1.65)	0.347
TKI treatment line (≥2nd-line vs 1st-line)	0.62 (0.39–0.99)	0.046	1.02 (0.69–1.50)	0.930
EGFR mutation subtype				
L858R vs 19-del	1.69 (1.34–2.12)	<0.001	–	–
Rare mutations vs 19-del	1.35 (0.91–2.01)	0.138	–	–
EGFR-TKI generation				
2nd vs 1st-generation	0.48 (0.32–0.73)	<0.001	–	–
3rd vs 1st-generation	0.73 (0.52–1.03)	0.077	–	–
Age (per 1-year increase)	1.00 (0.99–1.01)	0.836	0.99 (0.98–1.00)	0.074

[1] The PFS multivariate analysis applied stratified Cox regression, stratified by *EGFR* mutation subtype and EGFR-TKI generation; hence hazard ratios and *p*-values for stratified variables were not available (N/A). Statistically significant *p*-values (<0.05) are presented in bold.

Supplementary Table S2. Proportional-hazards assumption test based on Schoenfeld residuals for Cox regression models

Covariates	PFS-Stratified Cox[1]			OS-Standard Cox		
	χ^2	df	*p*-value	χ^2	df	*p*-value
HBsAg status	0.077	1	0.781	0.084	1	0.773
Age (per 1-year increase)	0.026	1	0.871	2.357	1	0.125
Sex	1.607	1	0.205	0.047	1	0.828
ECOG-PS	3.334	3	0.343	2.075	3	0.557
Smoking status	0.882	1	0.348	0.138	1	0.710
Lung cancer family history	2.117	1	0.146	0.323	1	0.570
TNM stage	–	–	–	0.285	1	0.593
Surgery	0.014	1	0.905	0.007	1	0.932
Radiotherapy	1.727	1	0.189	0.342	1	0.559
Prior chemotherapy alone	2.511	1	0.113	0.237	1	0.627
Liver metastasis	1.516	1	0.218	0.117	1	0.733
EGFR mutation subtype	–	–	–	2.466	2	0.291
EGFR-TKI generation	–	–	–	2.838	2	0.242
TKI line	0.208	1	0.648	1.368	1	0.242
Global test	16.230	13	0.237	14.615	18	0.688

[1]The PFS sensitivity model incorporated TKI treatment line as a covariate and was stratified by TNM stage, *EGFR* mutation subtype and EGFR-TKI generation. The OS sensitivity model incorporated TKI treatment line as a covariate without stratification. "–" indicates the variable was used for stratification and not tested for proportional-hazards assumption.

Supplementary Table S3. Baseline clinical characteristics of patients with primary T790M, L861Q, and G719-family *EGFR* mutations.

**Characteristic**	**G719 family** *n* **= 17**	**L861Q** *n* **= 12**	**Primary T790M** *n* **= 3**	***p*-value[1]**
Age, median [IQR]	67 [59, 75]	61 [51, 68]	59 [58, 68]	0.537
Sex, *n* (%)				0.776
Female	9 (53%)	5 (42%)	2 (67%)	
Male	8 (47%)	7 (58%)	1 (33%)	
HBsAg group				0.493
Negative	14 (72%)	8 (67%)	2 (67%)	
Positive	3 (18%)	4 (33%)	1 (33%)	
ECOG performance status, *n* (%)				0.651
0	10 (59%)	6 (50%)	2 (67%)	
1	5 (29%)	4 (33%)	1 (33%)	
2	2 (12%)	0 (0%)	0 (0%)	
Unknown	0 (0%)	2 (17%)	0 (0%)	
Smoking status, *n* (%)				0.859
Never-smoker	11 (65%)	8 (67%)	2 (67%)	
Smoker	6 (35%)	3 (25%)	1 (33%)	
Unknown	0 (0%)	1 (8.3%)	0 (0%)	
Lung cancer family history, *n* (%)				0.753
No	14 (82%)	10 (83%)	3 (100%)	
Yes	3 (18%)	1 (8.3%)	0 (0%)	
Unknown	0 (0%)	1 (8.3%)	0 (0%)	
TNM stage, *n* (%)				0.387
III	1 (5.9%)	3 (25%)	0 (0%)	
IV	16 (94%)	9 (75%)	3 (100%)	
Surgery, *n* (%)				0.048
Yes	16 (94%)	11 (92%)	1 (33%)	
No	1 (5.9%)	1 (8.3%)	2 (67%)	
Radiotherapy, *n* (%)				0.231
Yes	16 (94%)	9 (75%)	2 (67%)	
No	1 (5.9%)	3 (25%)	1 (33%)	

[1]P values were calculated using the Kruskal–Wallis rank-sum test for continuous variables and Fisher's exact test for categorical variables. No adjustment for multiple testing was performed.

Supplementary Table S4. Baseline clinical characteristics of patients with other uncommon *EGFR* mutations, including exon 19 insertions and isolated S768I.

**Characteristic**	**Exon 19 insertions** ***n*** **= 15**	**Isolated S768I** ***n*** **= 3**	**Other rare/unknown *EGFR* variants** ***n*** **= 25**
Age, median [IQR]	62 [48, 73]	57 [53, 64]	60 [52, 68]
Sex, *n* (%)			
Female	8 (53%)	2 (67%)	17 (68%)
Male	7 (47%)	1 (33%)	8 (32%)
HBsAg group			
Negative	13 (87%)	3 (100%)	21 (84%)
Positive	2 (13%)	0 (0%)	4 (16%)
ECOG performance status, *n* (%)			
0	7 (47%)	3 (100%)	11 (44%)
1	8 (53%)	0 (0%)	9 (36%)
2	0 (0%)	0 (0%)	3 (12%)
Unknown	0 (0%)	0 (0%)	2 (8.0%)
Smoking status, *n* (%)			
Never-smoker	12 (80%)	2 (67%)	18 (72%)
Smoker	3 (20%)	1 (33%)	6 (24%)
Unknown	0 (0%)	0 (0%)	1 (4.0%)
Lung cancer family history, *n* (%)			
No	14 (93%)	1 (33%)	23 (92%)
Yes	1 (6.7%)	1 (33%)	0 (0%)
Unknown	0 (0%)	1 (33%)	2 (8.0%)
TNM stage, *n* (%)			
III	2 (13%)	0 (0%)	2 (8.0%)
IV	13 (87%)	3 (100%)	23 (92%)
Surgery, *n* (%)			
No	15 (100%)	3 (100%)	25 (100%)
Yes	0 (0%)	0 (0%)	0 (0%)
Radiotherapy, *n* (%)			
No	14 (93%)	3 (100%)	21 (84%)
Yes	1 (7%)	0 (0%)	4 (16%)

Data are presented as n (%) for categorical variables and median [IQR] for continuous variables. No between-group statistical comparisons were performed.

Supplementary Table S5. Standardized mean differences (SMD) of baseline covariates before and after propensity score matching

**Covariate**	**SMD before PSM**	**SMD after PSM**
Demographic		
Age	0.832	0.166
Sex (Male)	0.131	0.104
ECOG performance status		
0	0.132	0.062
1	0.044	0.104
2	0.031	0.021
3	0.016	0.021
Unknown	0.040	0.000
Smoking status		
Never-smoker	0.044	0.083
Smoker	0.056	0.083
Unknown	0.012	0.000
Lung cancer family history		
No	0.049	0.062
Yes	0.023	0.062
Unknown	0.026	0.000
EGFR mutation type		
Exon 19 deletion	0.061	0.021
L858R	0.109	0.062
Rare mutations	0.032	0.062
Unknown subtype	0.017	0.021
EGFR-TKI generation		
First-generation	0.024	0.021
Second-generation	0.029	0.021
Third-generation	0.053	0.000
Tumor stage		
TNM stage IV	0.094	0.000
Anti-tumor treatment		
Surgery	0.226	0.000
Radiotherapy	0.529	0.000
Chemotherapy alone	0.132	0.021
Chemoimmunotherapy	0.052	0.000
Metastasis		
Liver metastasis	0.023	0.042
TKI line		
Second-line	0.054	0.021

SMD, standardized mean difference; PSM, propensity score matching. SMD values are presented as absolute values. An absolute SMD less than 0.1 was considered acceptable between-group balance. Variables with SMD > 0.1 after matching: Age (0.166), Sex (Male) (0.104), ECOG PS 1 (0.104).

Supplementary Table S6. Exploratory interaction *p*-values for HBsAg status with pre-specified clinical covariates in multivariate Cox regression models for overall survival and progression-free survival

**Pre-specified subgroups**	**Interaction *p* (OS)**	**Interaction *p* (PFS)**
Age	0.724	0.833
Sex	0.200	0.010
ECOG-PS	0.435	0.551
TNM stage	0.064	0.095
Liver metastasis	0.015	0.089
EGFR mutation subtype	$<$0.001	0.026
EGFR-TKI generation	0.006	$<$0.001

Interaction terms were incorporated into fully-adjusted multivariate Cox proportional-hazards models. Stratified subgroup Cox regression was not performed to avoid bias introduced by limited event counts. These analyses are exploratory; significant interaction *p*-values indicate heterogeneous prognostic effects of HBsAg status across subgroups and should be interpreted cautiously.

Supplementary Table S7. Baseline characteristics of HBsAg-positive *EGFR*-mutant NSCLC patients stratified by HBV-DNA test availability

**Variable**	**Overall(N=82)**	**HBV-DNA available (N=51)**	**HBV-DNA unknown (N=31)**	***p*-Value**
Age, median [IQR]	54.0 [36.0, 76.0]	56.0 [36.0, 74.0]	52.0 [40.0, 76.0]	0.180
Sex, *n* (%)				0.604
Male	44 (53.7)	29 (56.9)	15 (48.4)	
Female	38 (46.3)	22 (43.1)	16 (51.6)	
ECOG performance status, *n* (%)				0.164
0	48 (58.5)	29 (56.9)	19 (61.3)	
1	27 (32.9)	15 (29.4)	12 (38.7)	
2	6 (7.3)	6 (11.8)	0 (0.0)	
3	1 (1.2)	1 (2.0)	0 (0.0)	
Smoking history, *n*				0.408
Never-smoker	55 (67.1)	32 (62.7)	23 (74.2)	
Smoker	27 (32.9)	19 (37.3)	8 (25.8)	
Lung cancer family history, *n* (%)				0.193
No	76 (92.7)	49 (96.1)	27 (87.1)	
Yes	6 (7.3)	2 (3.9)	4 (12.9)	
EGFR mutation type,				0.217
Exon 19 deletion	43 (52.4)	28 (54.9)	15 (48.4)	
L858R	25 (30.5)	15 (29.4)	10 (32.3)	
Rare mutations	10 (12.2)	4 (7.8)	6 (19.4)	
Unknown subtype	4 (4.9)	4 (7.8)	0 (0.0)	
EGFR-TKI generation, *n* (%)				1.000
First-generation	56 (68.3)	35 (68.6)	21 (67.7)	
Second-generation	8 (9.8)	5 (9.8)	3 (9.7)	
Third-generation	18 (22.0)	11 (21.6)	7 (22.6)	
TNM stage, *n* (%)				0.182
III	14 (17.1)	6 (11.8)	8 (25.8)	
IV	68 (82.9)	45 (88.2)	23 (74.2)	
Surgery, *n* (%)				0.864
Yes	19 (23.2)	11 (21.6)	8 (25.8)	
No	63 (76.8)	40 (78.4)	23 (74.2)	
Radiotherapy, *n* (%)				1.000
Yes	47 (57.3)	29 (56.9)	18 (58.1)	
No	35 (42.7)	22 (43.1)	13 (41.9)	
Systemic therapy regimen, *n* (%)				0.683
No	59 (72.0)	38 (74.5)	21 (67.7)	
Chemo-only	23 (28.0)	13 (25.5)	10 (32.3)	

Baseline HBsAg quantification, *n* (%)				0.003
≤3000 IU/mL	60 (73.2)	31 (60.8)	29 (93.5)	
>3000 IU/mL	22 (26.8)	20 (39.2)	2 (6.5)	
Baseline HBeAg status, *n* (%)				0.645
Negative	77 (93.9)	47 (92.2)	30 (96.8)	
Positive	5 (6.1)	4 (7.8)	1 (3.2)	
Baseline liver function abnormality, *n* (%)				0.245
Normal	75 (91.5)	45 (88.2)	30 (96.8)	
Abnormal	7 (8.5)	6 (11.8)	1 (3.2)	
Baseline antiviral treatment, *n* (%)				<0.001
Yes	41 (50.0)	38 (74.5)	3 (9.7)	
No	41 (50.0)	13 (25.5)	28 (90.3)	
Baseline antiviral agent, *n* (%)				<0.001
No	41 (50.0)	13 (25.5)	28 (90.3)	
ETV	27 (32.9)	26 (51.0)	1 (3.2)	
TDF	3 (3.7)	3 (5.9)	0 (0.0)	
TAF	0 (0.0)	0 (0.0)	0 (0.0)	
LAM	10 (12.2)	8 (15.7)	2 (6.5)	
Others	1 (1.2)	1 (2.0)	0 (0.0)	

Supplementary Table S8. Clinical characteristics and management of HBV reactivation in *EGFR*-mutant lung adenocarcinoma patients treated with EGFR-TKI

ID	Age (y)/ Sex	*EGFR* mutation type	EGFR-TKI agent	TNM Stage	Baseline HBsAg (IU/mL)[a]	HBV antiviral regimen	Viral load (baseline → reactivation, IU/mL)	LFT change (baseline → highest during therapy, × ULN)[b]	Post-reactivation antiviral/liver-protective management	TKI treatment duration (m)	Time to HBV reactivation (m)	BOR	PFS（m）	OS (m)
1	68/F	Exon 19 deletion	Icotinib	IIIA	>3000	ETV	3780→13300	Normal→ 5.3×	Add ETV	11	5	SD	11.4	19.9
2	54/M	Exon 19 deletion	Icotinib	IVA	5042	ETV	400→ 7000	Normal→ 1.4×	Continue ETV	13	7	PR	11.8	25.9
3	52/M	Unkown	Osimertinib	IVB	658	None	Undetectable→183	2.6×→3.3×	LPA only	15	15	SD	12.6	30.5
4	48/M	Exon 19 deletion	Gefitinib	IVA	>3000	None	Undetectable→5820	Normal→ 2.0×	Add LAM	17	5	SD	17.6	62.2
5	48/F	L858R	Gefitinib	IVB	>3000	None	Undetectable→23500	Normal	None	18	10	SD	6	27.2

[a]Baseline HBsAg values exceeding the assay upper limit were documented as >3000 IU/mL.

[b]Liver function values (baseline and peak during therapy) were expressed as multiples of the upper limit of normal (× ULN), according to institutional laboratory reference ranges. Abnormal liver function was defined as ALT and/or AST > 1.0 × ULN.

Supplementary Table S9. Univariate and multivariable Cox regression analyses for progression-free survival in HBsAg-positive patients

Variable	Univariate HR (95% CI)	Univariate *p*-value	Multivariable HR (95% CI)	Multivariable *p*-value
Sex (Male vs Female)	1.96 (1.20–3.20)	0.007	1.64 (0.98–2.77)	0.061
Age (per 1-year increase)	0.99 (0.96–1.01)	0.305	–	–
ECOG performance status				
ECOG 1 vs 0	1.33 (0.81–2.19)	0.260	–	–
ECOG 2 vs 0	1.01 (0.40–2.57)	0.975	–	–
ECOG 3 vs 0	0.64 (0.09–4.70)	0.663	–	–
Smoking status (Smoker vs Never-smoker)	1.37 (0.84–2.25)	0.207	–	–
Family history of lung cancer (Yes vs No)	0.81 (0.35–1.89)	0.630	–	–
EGFR mutation subtype				
L858R vs Exon 19 deletion	1.23 (0.73–2.07)	0.441	–	–
Rare mutations vs Exon 19 deletion	0.93 (0.43–1.99)	0.849	–	–
Unknown subtype vs Exon 19 deletion	1.27 (0.45–3.58)	0.650	–	–
EGFR-TKI generation				
2nd-generation vs 1st-generation	0.36 (0.15–0.86)	0.022	0.43 (0.17–1.10)	0.079
3rd-generation vs 1st-generation	0.57 (0.31–1.05)	0.070	0.73 (0.37–1.44)	0.363
TNM stage IV vs III	0.61 (0.34–1.10)	0.101	0.84 (0.43–1.63)	0.606
Surgery (Yes vs No)	0.88 (0.52–1.50)	0.636	–	–
Radiotherapy (Yes vs No)	1.38 (0.87–2.20)	0.167	–	–
Systemic therapy (Yes vs No)	1.20 (0.72–2.00)	0.494	–	–
Liver metastasis (Yes vs No)	1.67 (0.79–3.53)	0.182	–	–
Prior antiviral drug (vs None)				
ETV vs None	1.10 (0.66–1.84)	0.712	1.09 (0.63–1.89)	0.764
TDF vs None	0.35 (0.08–1.48)	0.155	0.44 (0.10–2.02)	0.289

Variable	Univariate HR (95% CI)	Univariate *p*-value	Multivariable HR (95% CI)	Multivariable *p*-value
LAM vs None	1.51 (0.75–3.04)	0.252	1.12 (0.49–2.56)	0.783
Other vs None	28.33 (2.91–275.82)	0.004	19.52 (1.90–200.61)	0.012
HBV reactivation (Yes vs No)	0.94 (0.34–2.59)	0.903	0.83 (0.29–2.33)	0.719
Baseline HBV-DNA load (vs Unknown)				
Low/negative vs Unknown	0.79 (0.48–1.28)	0.335	–	–
High vs Unknown	0.67 (0.30–1.46)	0.312	–	–
Baseline liver function abnormality (ALT >40 vs Normal)	1.44 (0.62–3.37)	0.399	–	–
TKI line (Second-line vs First-line)	2.33 (1.20–4.53)	0.013	1.85 (0.87–3.96)	0.112

Supplementary Table S10. Univariate and multivariable Cox regression analyses for overall survival in HBsAg-positive patients

Variable	Univariate HR (95% CI)	Univariate *p*-value	Multivariable HR (95% CI)	Multivariable *p*-value
Sex (Male vs Female)	1.68 (1.05–2.70)	0.032	1.54 (0.93–2.53)	0.091
Age (per 1-year increase)	1.00 (0.97–1.02)	0.718	–	–
ECOG performance status				
ECOG 1 vs 0	1.12 (0.68–1.84)	0.667	–	–
ECOG 2 vs 0	1.27 (0.48–3.32)	0.633	–	–
ECOG 3 vs 0	1.39 (0.19–10.17)	0.748	–	–
Smoking status (Smoker vs Never-smoker)	1.52 (0.93–2.48)	0.095	–	–
Family history of lung cancer (Yes vs No)	0.63 (0.25–1.60)	0.335	–	–
EGFR mutation subtype				
L858R vs Exon 19 deletion	1.41 (0.84–2.39)	0.197	–	–
Rare mutations vs Exon 19 deletion	1.51 (0.72–3.15)	0.271	–	–
Unknown subtype vs Exon 19 deletion	2.03 (0.72–5.76)	0.183	–	–
EGFR-TKI generation				
2nd-generation vs 1st-generation	0.31 (0.11–0.87)	0.026	0.28 (0.09–0.83)	0.021
3rd-generation vs 1st-generation	0.97 (0.54–1.73)	0.911	0.94 (0.47–1.87)	0.861
TNM stage IV vs III	0.73 (0.40–1.31)	0.288	0.87 (0.43–1.73)	0.682
Surgery (Yes vs No)	0.85 (0.48–1.50)	0.568	–	–
Radiotherapy (Yes vs No)	0.85 (0.53–1.36)	0.502	–	–

**Variable**	**Univariate HR (95% CI)**	**Univariate *p*-value**	**Multivariable HR (95% CI)**	**Multivariable *p*-value**
Systemic therapy (Yes vs No)	1.23 (0.74–2.05)	0.426	–	–
Liver metastasis (Yes vs No)	2.73 (1.33–5.62)	0.006	3.84 (1.65–8.94)	0.002
Prior antiviral drug (vs None)				
ETV vs None	0.87 (0.51–1.48)	0.602	1.00 (0.55–1.81)	0.989
TDF vs None	0.52 (0.13–2.18)	0.374	0.65 (0.15–2.85)	0.568
LAM vs None	1.09 (0.54–2.21)	0.805	0.79 (0.34–1.83)	0.582
Other vs None	5.42 (0.70–41.92)	0.105	5.21 (0.61–44.17)	0.131
HBV reactivation (Yes vs No)	1.89 (0.75–4.72)	0.175	1.29 (0.49–3.40)	0.614
Baseline HBV-DNA load (vs Unknown)				
Low/negative vs Unknown	0.76 (0.46–1.25)	0.285	–	–
High vs Unknown	0.84 (0.38–1.84)	0.665	–	–
Baseline liver function abnormality (ALT >40 vs Normal)	2.66 (1.18–5.97)	0.018	–	–
TKI line (Second-line vs First-line)	1.58 (0.85–2.94)	0.152	1.54 (0.70–3.41)	0.283
